# Breastfeeding Duration and Early Parenting Behaviour: The Importance of an Infant-Led, Responsive Style

**DOI:** 10.1371/journal.pone.0083893

**Published:** 2014-02-12

**Authors:** Amy Brown, Bronia Arnott

**Affiliations:** 1 Department of Public Health and Policy Studies, Swansea University, Swansea, United Kingdom; 2 Department of Psychology, Durham University, Durham, United Kingdom; Indiana University, United States of America

## Abstract

**Background:**

Popular parenting literature promotes different approaches to caring for infants, based around variations in the use of parent-led routines and promoting infant independence. However, there is little empirical evidence of how these early behaviours affect wider parenting choices such as infant feeding. Breastfeeding often requires an infant-led approach, feeding on demand and allowing the infant to regulate intake whilst conversely formula feeding is open to greater caregiver manipulation. The infant-led style associated with breastfeeding may therefore be at odds with philosophies that encourage strict use of routine and independence. The aim of this study was to explore the association between early parenting behaviours and breastfeeding duration.

**Methods:**

Five hundred and eight mothers with an infant aged 0–12 months completed a questionnaire examining breastfeeding duration, attitudes and behaviours surrounding early parenting (e.g. anxiety, use of routine, involvement, nurturance and discipline). Participants were attendees at baby groups or participants of online parenting forums based in the UK.

**Results:**

Formula use at birth or short breastfeeding duration were significantly associated with low levels of nurturance, high levels of reported anxiety and increased maternal use of Parent-led routines. Conversely an infant-led approach characterised by responding to and following infant cues was associated with longer breastfeeding duration.

**Discussion:**

Maternal desire to follow a structured parenting approach which purports use of Parent-led routines and early demands for infant independence may have a negative impact upon breastfeeding duration. Increased maternal anxiety may further influence this relationship. The findings have important implications for Health Professionals supporting new mothers during pregnancy and the postpartum period.

## Introduction

The importance of breastfeeding for infant and maternal health is well established. Infants who are formula fed are at increased risk of gastrointestinal and respiratory disorders, allergies and obesity [1], [2]. Despite this, levels of breastfeeding in the UK are low [3]. Although physiological contraindications to breastfeeding do exist [4], social and psychological variables are known to play a large role in affecting breastfeeding duration [5]. Beliefs that breastfeeding is inconvenient and difficult or that formula-fed infants are more content impact on how a mother adapts to breastfeeding [6]. Physical difficulties and lack of support can exacerbate difficulties [5], [7]. Moreover, low confidence, poor self efficacy and anxiety can also lead to formula use [8], as well as concerns for infant health, growth and development [9], [10].

However, little empirical evidence exists in relation to how early infant feeding fits within wider parenting behaviours and contexts. Previous research exploring the conceptualisation and influence of parenting styles for older children typically categorised behaviour along two dimensions of warmth and control [11]. Authoritative parents typically respond in a warm and responsive way to their child's signals but also exert an appropriate amount of age appropriate control over their child's behaviour. Conversely, authoritarian parents exhibit high levels of control combined with a low level of nurturance. Finally, permissive parents combine low levels of control with high warmth [12]. In general, an authoritative approach has been associated with desired outcomes including positive child conduct [13], enhanced cognitive development [14], and healthier nutritional intake and weight-gain trajectories [15]. How these elements of parenting older children might apply to parenting an infant and their impact upon the infant are however unclear.

One central question appears to be how aspects of parenting styles, particularly around control and nurturance might affect or drive decision to breast or formula feed. To be successful, breastfeeding often has to be baby-led, following the infants cues of hunger and satiety to promote milk supply [16]. Breastfed infants typically feed more frequently and irregularly than formula fed infants as breast milk is easily digested and varies in energy density over the course of the day [17]. On demand, unrestricted breastfeeding is associated with an earlier onset of milk production [18], faster regain of birth weight [19] and a reduced occurrence of issues related to breastfeeding discontinuation such as nipple soreness and engorgement [20]. Conversely formula feeding can be open to greater caregiver manipulation as it is easier to perform to a parent-led schedule [18]. The baby-led nature of breastfeeding can however evoke maternal anxiety regarding infant consumption or clash with maternal desire for control and predictability [10]. Essentially, breastfeeding may ‘conflict’ with overall approach to care giving.

In an initial study we characterised variations in parenting style during infancy exploring developed concepts of nurturance and control but applying them to infant care. For example, issues such as use of parent-led routine and encouraging the infant to self-settle were explored [21]. Here we examine the association between these early parenting behaviours and breastfeeding duration.

## Methodology

### Participants

All aspects of this study were performed in accordance with the ethical standards set out in the 1964 Declaration of Helsinki. Ethics approval was granted by Swansea University (UK) Department of Psychology Research Ethics Committee.

Five hundred and eight mothers with an infant aged between 0 and 12 months (mean age 27.47 weeks) (SD: 15.02) completed a self report questionnaire. Participants were recruited from local mother and baby groups in Swansea and Durham and through online parenting forums based in the UK. The groups were located in areas of varying deprivation as measured by the Welsh and English Indices of Multiple Deprivation. Groups were both privately run or based in Children's Centres or Family and Community Centres, encouraging a wide range of participation in terms of demographic background. For the groups, contact was made with group leaders who distributed questionnaires to group members. Questionnaires were returned to the leader in a sealed envelope or via post to the researcher. Questionnaires had information letters attached with details of how to contact the researcher if further information was required.

Study adverts were also placed on specific research request boards on online message boards on parenting forums based in the UK (e.g. www.mumsnet.com; www.bounty.com) with an online link to complete the questionnaire via survey monkey. All participants were based in the UK and this was verified by provision of UK postcode. Details were given as above for how to contact the researcher if needed. Questionnaire completion via either method implied consent.

Participants completing the questionnaire via paper or online copy were given a written debrief at the end of the questionnaire and given researcher details to contact if they wanted further information. All participants were given instruction to contact their relevant health professional if completing the questionnaire had raised any questions or issues with regard to caring for their baby.

### Measures

The self report questionnaire explored maternal demographic background (age, education, parity), infant details (age, gender, birth weight) and details regarding breastfeeding initiation, duration and exclusivity. Participants indicated whether they breast or formula fed at birth, duration of breastfeeding if they had stopped and timing of introduction to use of formula and complementary foods if appropriate.

Participants also responded to a series of items examining their approach to parenting their infant (Infancy Parenting Styles Questionnaire (IPSQ) [21]. Items examined maternal involvement, responsiveness, sensitivity and discipline which are similar to those found in literature relating to parenting older children alongside more age specific behaviours of maternal anxiety and concern for her infant and maternal involvement in infant development such as concern for weight gain, sleep and early development (Table 1). The IPSQ uses a five point likert scale for response (strongly agree to strongly disagree) with a a higher score on each factor indicating stronger agreement.

**Table 1:** Infant Parenting styles Questionnaire factors, items and mean scores.

*Factor*	*Items*
Discipline	You can spoil a baby
	My baby needs to learn the difference between right and wrong
	It is never too young to start disciplining a child
	Sometimes my baby cries to try and manipulate me
	My baby sometimes does things that are naughty
	Babies under one year do not need discipline
Parent-led routine	I have a strict day to day routine for my baby
	Babies need a routine
	People who don't use a routine make a rod for their own back
	Everyone is happiest when the baby is in a routine
	My baby sets their own routine
	A routine makes a baby calm and secure
Anxiety	I regularly ask other people advice about my baby's behaviour
	I worry a lot about my baby
	I regularly seek advice from my health visitor/GP about my baby
	I often check baby books to see if my baby is on target
Nurturance	Babies should be encouraged to entertain themselves
	I make sure I put my baby down regularly
	Cuddling babies all the time makes them too dependent
	I generally like to keep my baby as close as possible to me
Involvement	I encourage my baby to develop skills such as walking or talking
	I do lots of organised activities with my baby
	I make sure I play, read or sing with my baby very regularly
	Babies need lots of parental input such as reading and activities
	It is very important my baby meets developmental milestones

### Data analysis

Distribution of breastfeeding duration was non-normal (Kolmogorov-Smirnov = .278 = .001) with a high proportion of mothers ceasing breastfeeding in the first few days and weeks or breastfeeding for a longer duration [as reflected in many studies such as the UK Infant Feeding Survey [3]. Therefore breastfeeding duration data was transformed and the natural logarithms computed used in order to correct for the skewed distribution. Mean scores for each factor were computed for the IPSQ [21].

Factor scores were compared using MANCOVA for infant feeding method at birth [breast/formula] and any breast milk at two, six, twelve and twenty six weeks. Maternal factors (age, education, parity, return to work), infant factors (infant age, birth weight) and birth experience (gestation, mode (section versus vaginal birth) were controlled for.

## Results

The mean age of the respondents was 30.72 (SD: 5.12) years, (range from 17 to 44) and the mean number of years in education was 14.61 (SD: 2.53) (range from 12 to 20 years). Infant age ranged from 0 and 12 months (mean age 27.47 weeks (SD: 15.02). 29.4% of mothers were primiparous with the majority (54.1%) multiparas with two children. No significant difference was seen in mean age, years in education, marital status, breastfeeding duration or parenting behaviours between mothers who completed a paper or online version of the questionnaire. Exclusion criteria included multiple birth, low birth weight (<2500 g) or premature birth (<37 weeks). Demographic spread can be found in Table 2.

**Table 2:** Sample distribution by Demographic Factors.

Indicator	Group	N	%
Age	≤19	15	3.0
	20–24	55	10.5
	25–29	137	27.0
	30–34	179	35.6
	35≥	119	23.4
Education	School	142	27.9
	College	115	22.3
	Higher	251	49.8
Marital Status	Married	279	54.9
	Cohabiting	209	41.2
	Single	18	3.6
	Widowed	2	0.2
Maternal Occupation	Professional & Managerial	112	22.0
	Skilled	194	38.4
	Unskilled	78	15.4
	Unemployed	21	4.1
	Stay at home parent	103	20.3

### Parenting style

Significant associations were found between parenting style and maternal and infant characteristics (Table 3). Mothers who were older and more educated were significantly more likely to report higher levels of anxiety and routine whilst reporting lower levels of nurturance, whilst older mothers were also significantly more likely to use a parent-led routine. Age of infant was also significantly associated with increased use of a parent-led routine and discipline and also lower levels of nurturance. Finally, birth weight was significantly inversely associated with anxiety and positively associated with greater use of parent-led routine and discipline. As noted above these variables were controlled for throughout the analyses.

**Table 3:** Association between maternal and infant characteristics and parenting behaviours.

	Discipline	Parent-led routine	Anxiety	Nurturance	Involvement
Maternal Age	−.23	.126**	.089*	−.130**	−.044
Maternal education	.82	.223**	.094*	−.355**	.056
Age of infant	.044	.113**	.012	−.097*	.069
Birth weight	.096*	.113**	−.095*	.032	−.071

Pearson's correlations:

* = p<0.05;

** = p<0.01.

### Mode of feeding at birth and parenting style

Mothers indicated whether they breastfed (n = 400), gave expressed milk (n = 30) or formula fed (n = 78) at birth. A significant difference on the parent led routine factor (F (1, 505) = 4.888, p = 0.008) was found for feeding method at birth. Post hoc Bonferroni tests showed that mothers who breastfed or expressed breast milk at birth later reported using significantly lower levels of parent-led routine than mothers who formula fed. No significant difference was found between those who breastfed directly or gave expressed milk.

Significant differences in infant feeding were also found for the anxiety factor (F (1, 505) = 2.180, p = .006). Mothers who breastfed at birth scored significantly lower on this factor compared to mothers who fed expressed milk or formula fed. No significant difference was found between those who fed expressed milk or gave formula.

Scores on the nurturance factor also varied significantly for method of feeding at birth (F (1, 505) = 4.537, p = .011). Mothers who breastfed or expressed breast milk at birth later reported significantly higher scores on this factor compared to mothers who formula fed.

No significant difference was found in method of feeding at birth and scores on discipline or Involvement factors.

### Breastfeeding duration and parenting style

The association between breastfeeding duration and parenting styles was also examined. The five parenting factors were compared for infants who were receiving any breast milk (or no breast milk) at two, six, twelve and twenty six weeks (Table 4). As infants in the sample were aged 0–12 months, at each stage the sample was reduced in size to infants that age or older.

**Table 4:** Breastfeeding and parenting behaviours: Showing mean scores (and standard deviation) at specific time points postpartum.

Time post partum	Any Breastfeeding	N	Discipline	Parent-led routine	Anxiety	Nurturance	Involvement
Birth	Yes	430	3.52 (.61)	2.90 (.52)**	3.37 (.73)**	2.9 (.43)*	2.36 (.34)
	No	78	3.51 (.63)	3.26 (.53)**	3.89 (.81)**	2.91 (.52)*	2.31 (.33)
Two weeks	Yes	333	3.50 (.61)	2.79 (.52)**	3.10 (.74)**	3.45 (.52)**	2.29 (.33)
	No	143	3.57 (.68)	3.26 (.54)**	3.47 (.35)**	3.01 (.67)**	2.28 (.35)
Six weeks	Yes	246	3.49 (.60)	2.75 (.52)**	3.19 (.75)*	3.48 (.56)**	2.38 (.32)
	No	170	3.56 (.67)	3.10 (.55)**	3.44 (.69)*	3.12 (.48)**	2.33 (.36)
Twelve weeks	Yes	155	3.45 (.61)	2.87 (.49)*	3.22 (.69)	3.24 (.42)	2.38 (.30)
	No	221	3.52 (.64)	3.21 (.43)*	3.27 (.62)	3.18 (.58)	2.33 (.36)
Twenty six weeks	Yes	77	3.49 (.59)	2.89 (.52)*	3.25 (.74)	3.15 (.62)	2.38 (.31)
	No	212	3.53 (.63)	3.15 (.63)*	3.28 (.63)	3.10 (.43)	2.34 (.35)

MANOVA:

* = p<0.05;

** = p<0.01.

Breastfeeding duration was also significantly associated with parenting styles. Mothers who were giving any breast milk at two weeks scored significantly lower on parent-led routine (F (1, 472) = 9.813, p = .002) and anxiety (F (1, 472) = 6.632, p = .010) and significantly higher on nurturance (F (1, 472) = 10.779, p = .001) compared to mothers who were giving no breast milk.

Similarly for any breastfeeding at six weeks, mothers who were giving any breast milk scored significantly lower on parent-led routine (F (1, 409) = 10.552, p = .001) and anxiety (F (1, 409) = 5.476, p = .020) and significantly higher on nurturance (F (1, 409) = 12.415, p = .000) compared to mothers who were giving no breast milk.

Differences in parent-led routine remained significant for any breastfeeding at twelve (F (1, 359) = 5.122, p = .024) and twenty six weeks (F (1, 359) = 5.263, p = .022). However significant differences were no longer found for anxiety or nurturance.

No significant difference was found at any stage for discipline or involvement for milk feeding group.

## Discussion

This paper examined the association between variations in approaches to parenting in early infancy and breastfeeding initiation and duration. Overall, parenting approaches that were higher in parent-led routine and anxiety and lower in nurturance were associated with a decreased likelihood that the infant was breastfed at birth and an increased likelihood that formula was introduced within the first six weeks postpartum. Given the importance of breastfeeding for infant and maternal health [1], understanding the impact and interaction of parenting styles upon breastfeeding is an important element in understanding maternal motivation to initiate and barriers to continuation.

Mothers who reported that they used high levels of parent-led routine day to day for their infant were less likely to initiate or continue breastfeeding. This relationship could be bidirectional. Mothers may adapt their use of routine to their chosen feeding method. Breastfeeding typically needs to baby-led and on demand to establish milk supply [18] whereas formula feeding can encourage a stricter feeding schedule and routine [6]. Moreover as breastfed infants often feed more frequently and irregularly compared to formula fed infants, a parent-led routine can be more difficult to establish [10]. Potentially mothers who breastfeed learn to, or have to, adopt a baby-led feeding pattern to continue breastfeeding whereas mothers who formula feed learn to, or choose to, follow a stricter parent-led routine. Alternatively, maternal desire for routine may drive infant feeding choice. Mothers who wish to have a strict routine may choose to formula feed for this reason. Indeed the perceived irregularity, commitment and hassle of breastfeeding are commonly cited by pregnant women for deciding not to breastfeed or to stop after a short duration of time [5]. Either way, a strict parent-led routine for infant feeding may not be beneficial to infant health, either through discouraging breastfeeding or because encouraging a baby to feed to a parent-led routine rather than its own natural patterns may promote obesity [6].

Breastfeeding initiation and continuation in the early weeks were associated with higher scores on the Nurturing factor, which included behaviours such as keeping the infant as close as possible. Nurturing behaviour is associated with improved breastfeeding outcomes; keeping the infant close, including skin-to-skin contact as soon after the birth is believed to play a critical role in breastfeeding initiation and duration [22]. Responding promptly to the infants signals of hunger encourages prolactin production which is essential to milk production [23] which is more likely if the infant is kept in close proximity including behaviour such as room sharing [24]. Again this relationship may be bidirectional – breastfeeding may encourage the mother to keep her infant close to her whereas perhaps formula feeding, where feeds are less frequent, may promote greater independence. Conversely, desire for nurturance may drive feeding choice; Mothers may choose to formula feed as they wish to have greater independence from their infant, allowing others to feed the infant or through the need to balance other responsibilities [5], [8]. Formula milk is perceived to promote infant sleep and encourage the infant to settle [10], potentially due to the increased time it takes to digest feeds [17]. An independent infant can often be viewed as a measured of parenting success e.g. ‘a good baby’ [25]. Thus for those who desire independence, breastfeeding may be incompatible.

Finally, greater maternal anxiety factor scores were associated with using formula from birth or stopping breastfeeding within the first six weeks. Mothers scoring high on this factor reported greater anxiety regarding their infants' development and greater tendency to seek advice from others [21]. The association between maternal anxiety and formula use has been well established. Mothers who report low self-efficacy and confidence are more likely to choose to formula feed [7]. Specific anxieties related to poor weight gain [9], concern regarding milk production [8], or that the infant is not receiving enough milk [10] are all linked to a shorter breastfeeding duration; often shorter than the mother desired. Formula feeding may provide reassurance for a mother with higher anxiety as intake is far more measurable; the amount consumed is visible, nutrient content listed on the tin and direct instructions about quantities to offer [18]. Conversely, it is possible that a negative experience of breastfeeding, leading to formula use, may increase overall maternal anxiety for their infant.

One potential insight into the development of parenting styles across this time is the sub sample of mother who gave expressed milk rather than breastfeeding directly at birth. In the UK mothers are entitled to up to nine months of paid maternity leave [26] which gives them opportunity to breastfeed for a number of months before they return to work. Low numbers of mothers in the UK choose to exclusively express milk for their infant rather than directly breastfeed [3]. This is in contrast to countries such as the USA where mothers often return to work soon after the birth, leading to greater numbers of mothers choosing to express milk for their infants for this reason. In the UK, when mothers choose to express, it is usually due to complications surrounding the birth such as infant or maternal health difficulties and/or low infant birth weight lead to the infant being given expressed milk rather than fed directly [27]. However, a negative birth experience can have a major impact on a new mother. It can increase maternal anxiety for her infant [28] that can last into childhood [29]. Concerns regarding growth and milk supply are especially strong [30]. Here, mothers who expressed breast milk at birth reported the highest levels of anxiety regarding their infant suggesting that early parenting styles may be affected by significant experiences surrounding the birth and first year. Again, further research is needed to ascertain how parenting styles form and develop.

A key question is the potential impact of the findings for those working to support breastfeeding mothers. There is a clear message emerging from the data that maternal beliefs and behaviours regarding wider parenting approach during infancy are associated with, and may be affecting, milk feeding choice. In the UK there is a very profitable market of baby care books aimed at parents of young infants [31]. These books often promote specific approaches to caring for an infant, typically focussed around how the parent should respond to the infant and use of parent led routines for sleep and feeding. Generally these books fall into two categories; those proposing a parent-led approach whereby strict sleep and feeding routines are imposed and those which are more baby-led encouraging the parent to respond to and follow infant cues and rhythms [32]. However, these books are typically not evidence based due to the lack of research exploring outcomes in this area. Given the popularity of parenting manuals that might promote structured methods of interaction and approach to the infant [21], [32], greater awareness needs to be raised as to how these books may be influencing maternal behaviour and choices, especially due to the lack of underpinning empirical evidence.

Moreover awareness should also be given by Health Professionals as to the influences beliefs about infant care approaches might have upon breastfeeding duration. Beliefs regarding routine and interaction with their infant or misconceived ideas about what normal infant sleep or feeding behaviour should be like might affect their parenting choices. Although the data presented here is correlational, given the association between infant feeding choice at birth and parenting style, perhaps the discussion of these antenatally may be important.

One specific application for health professionals could be the notable differences in both anxiety and nurturance between breast and formula groups up to six weeks postpartum but that were not significant after this time. Breastfeeding in the early days can be demanding but it is often viewed as established and easier after the infant reaches six weeks old [33], [34]. Health professionals might consider this with mothers who are feeling anxious about breastfeeding or concerned at how close they will need to remain to the infant at all times after the birth. High numbers of mothers initiate breastfeeding at birth but there is a high drop of rate in continuation, often before the mother was planning to stop breastfeeding [3]. Discussing with mothers how breastfeeding may change and become easier and less consuming after the first few weeks might encourage initiation or duration rates.

Despite the potential impact of a parent-led style upon breastfeeding duration, wider consideration does need to be given to the overall parenting experience. Sleep deprivation [35] and infant crying [36] are associated with increased risk of postnatal depression and maternal feelings of loss of control or identity [37]. An infant-led approach has been linked to night wakings for longer [38] whilst parent-led sleep training was related to reduced incidence of postnatal depression [39]. At an individual level a parent-led style and bottle feeding may have benefits that overall contribute to a more positive outcome for the family [40]. However, given the considerable importance of breastfeeding for both infant and maternal health [3], [4], more emphasis should be placed on offering increased maternal practical and emotional support and boosting confidence to enable her to breastfeed [41], [42]. Educating partners, grandparents and the wider public to the importance of following infant cues may play a key role here as might increased funding for breastfeeding peer supporters or family support workers.

There are limitations to the study. Firstly, as discussed above the data are correlational, and cannot imply cause and effect. It is possible that parenting styles influence feeding choices. On the other hand, it is possible that infant feeding choices shape parenting style. Alternatively it may be that the relationship between the variables is additive. Further although this exploratory study did include a wide range of potentially mediating variables it may be that there are other, as yet untested variables, which could explain the association. A longitudinal study exploring how attitudes and behaviors develop and change over time would address these issues. Related to this, research could consider whether these attitudes are malleable and whether interventions to promote an infant-led parenting approach may increase breastfeeding initiation and duration.

Secondly, the sample was self selecting with a slightly higher than average number of women who initiated breastfeeding at birth. Although responses were received from a variety of demographic backgrounds, the sample was older with a higher level of education than the UK average [43] with 92% of the sample of White British Origin limiting the generalisability of the findings. This was an exploratory study and further research is needed to test the findings in a population based sample to examine whether the structure and reliability of the questionnaire remains for a wider demographic. Moreover, potentially mothers with a higher level of education are more likely to research and read about their parenting choices which may affect their exposure to early parenting books and thus attitudes and behaviors e.g. decision to use a parent–led routine. Mothers with a more deprived background however are more likely to seek the guidance and support of family, particularly their mother, who may have generational ideas about how infants should be cared for [44]. Future research could ask parents to report sources of information that they have consulted regarding parenting behaviors and attitudes.

Thirdly, seventy two per cent of the responses were collected via the online survey link which could be considered a limitation [45]. However, the internet is now widely used, especially amongst pregnant and new mothers [46] and this was seen in the demographic spread of those completing online. Online recruitment methods are now increasing in popularity in health research [47], [48], [49]. No significant differences were seen in responses collected online or via paper copy. For an initial exploration of this under researched area, utilising internet recruitment methods allowed this new data to be collected. Further research should however now use more widely accessible methods in population based samples.

In conclusion, the findings raise pertinent questions in relation to the impact of parenting styles during early infancy upon breastfeeding initiation and duration. The main tenet that maternal desire for Parent-led routine and infant independence, and mothers' anxiety can impact negatively upon breastfeeding is an important consideration for those working to support pregnant and new mothers in the postnatal period. Moreover, given the popularity of specific approaches to early parenting in the popular literature, further research is needed to examine how these might be impacting upon infant health and development. Awareness needs to be raised about how the promotion of parent-led parenting styles might impede breastfeeding initiation and duration.
